# Proteins and Transcriptional Dysregulation of the Brain Extracellular Matrix in Parkinson’s Disease: A Systematic Review

**DOI:** 10.3390/ijms24087435

**Published:** 2023-04-18

**Authors:** Wote Amelo Rike, Shani Stern

**Affiliations:** Sagol Department of Neurobiology, Faculty of Natural Sciences, University of Haifa, Haifa 3498838, Israel; wotepharma@yahoo.com

**Keywords:** Parkinson’s disease, extracellular matrix, post mortem tissue, induced pluripotent stem cells, proteins, transcription

## Abstract

The extracellular matrix (ECM) of the brain is a dynamic structure made up of a vast network of bioactive macromolecules that modulate cellular events. Structural, organizational, and functional changes in these macromolecules due to genetic variation or environmental stressors are thought to affect cellular functions and may result in disease. However, most mechanistic studies to date usually focus on the cellular aspects of diseases and pay less attention to the relevance of the processes governing the dynamic nature of the extracellular matrix in disease pathogenesis. Thus, due to the ECM’s diversified biological roles, increasing interest in its involvement in disease, and the lack of sufficient compiled evidence regarding its relationship with Parkinson’s disease (PD) pathology, we aimed to compile the existing evidence to boost the current knowledge on the area and provide refined guidance for the future research. Here, in this review, we gathered postmortem brain tissue and induced pluripotent stem cell (iPSC)-related studies from PubMed and Google Scholar to identify, summarize and describe common macromolecular alterations in the expression of brain ECM components in Parkinson’s disease (PD). A literature search was conducted up until 10 February 2023. The overall hits from the database and manual search for proteomic and transcriptome studies were 1243 and 1041 articles, respectively. Following a full-text review, 10 articles from proteomic and 24 from transcriptomic studies were found to be eligible for inclusion. According to proteomic studies, proteins such as collagens, fibronectin, annexins, and tenascins were recognized to be differentially expressed in Parkinson’s disease. Transcriptomic studies displayed dysregulated pathways including ECM–receptor interaction, focal adhesion, and cell adhesion molecules in Parkinson’s disease. A limited number of relevant studies were accessed from our search, indicating that much work remains to be carried out to better understand the roles of the ECM in neurodegeneration and Parkinson’s disease. However, we believe that our review will elicit focused primary studies and thus support the ongoing efforts of the discovery and development of diagnostic biomarkers as well as therapeutic agents for Parkinson’s disease.

## 1. Introduction

Parkinson’s disease (PD) is an extremely heterogeneous neurodegenerative disorder characterized by the cardinal features of the hallmark presence of bradykinesia, rest tremor, and rigidity [1,2]. These motor signs are often preceded by non-motor manifestations such as constipation, autonomic and olfactory dysfunction, sleep disturbances, depression, and anxiety. This motor dysfunction is due, in a large part, to the loss of dopamine (DA)-containing neurons in the substantia nigra pars compacta (SNc), a part of the midbrain that plays an important role in the regulation of movement [3,4,5]. It has been estimated that, at clinical presentation, more than 60% of SNc DA neurons have already degenerated, and there is also an 80% reduction in dopamine content in the striatum [3,4,6]. Although the disease appears multifactorial in origin, it could result from a complex interaction between genetics and the environment, and commonly affects older people, coming in second only to Alzheimer’s disease in neurodegenerative diseases [7]. Several genes or loci including synuclein alpha (*SNCA*), leucine-rich repeat kinase 2 (*LRRK2*), Parkin RBR E3 ubiquitin protein ligase (*PARKIN*), PTEN-induced kinase 1 (*PINK1*), and glucosylceramidase beta 1 (*GBA1*), which have a genetic and neuropathologic link to PD, have been identified as being involved in PD, have been reviewed elsewhere [8,9,10,11,12] and are also registered in the Online Mendelian Inheritance in Man (OMIM) database. There has been speculation that extracellular alpha-synuclein may contribute to the spread of PD by activating microglia and causing neuroinflammation [13]. This activation of microglia could also have significant implications for the extracellular matrix (ECM) [14]. As a result of the aging population and the world’s increasing industrialization, which is linked to environmental risk factors, the prevalence of PD is expected to rise steadily to around 13 million by 2040 [15]. The late onset of motor symptoms, after the loss of the majority of dopaminergic neurons, and the lack of any reliable biomarkers is the current diagnostic challenge of the early detection of and intervention in ever-increasing PD cases [16]. Thus, the discovery and development of specific biomarkers for early diagnosis and neuroprotective strategies are of utmost importance for this currently incurable disease.

The ECM is a three-dimensional, cell-secreted, ubiquitous, and complex macromolecular network of proteins and glycans, which is built up around the cellular components of every tissue [17,18]. The ECM demonstrates great tissue specificity due to its varied compositions and topographies that are formed by a dynamic interaction between the numerous cells in each tissue and the altering milieu [19]. Brain ECM networks account for 10–20% of brain volume and constitute collagenous and non-collagenous proteins, glycoproteins, hyaluronan, and proteoglycans (PGs) [20,21]. The network also binds secreted proteins such as growth factors and is known to interact with numerous cell surface receptors, such as integrins, thereby providing biochemical cues that regulate the activities of protein complexes and mediate cell-to-cell communication [22,23]. The intricate chemical composition enables the ECM to play a crucial role in governing essential cellular behaviors and characteristics such as cell survival, function, attachment, and proliferation, and it also aids in the physical organization of neuronal and glial cells into distinct brain areas [24,25].

While the ECM’s composition is broadly similar across different tissues, there are notable differences in the types and amounts of molecules present in the ECM of different organs. In the brain, the ECM is designed to maintain homeostatic processes that are crucial for the survival of terminally differentiated cells, which generally do not regenerate [26,27]. Functionally, the ECM surrounding blood vessels in the brain, which includes basement membrane components such as laminin and collagen IV, is more similar to the systemic ECM. However, closer to the brain’s cellular environment, the ECM has different anatomies such as the diffuse interstitial matrix and the condensed perineuronal nets (PNNs) that surround specific populations of neurons [28]. The ECM is even specific around the synaptic elements [29], further stressing its importance to brain function. In these specialized structures, the brain ECM is distinct and plays an important role in regulating neuronal activity and synaptic plasticity [30,31,32,33,34]. As a result, it has a unique molecular makeup, with a long chain of hyaluronic acid as its primary component [28]. Additionally, the components are spatially and temporally controlled throughout brain development [26,27]. As an individual reaches adulthood, the composition of the brain’s ECM mainly consists of glycans and proteoglycans, with fewer amounts of collagen and other fibrillar ECM proteins, but it still serves to anchor different structures and guard against abnormal synaptic remodeling [30,35]. Its components are, however, poorly regulated in certain diseases, and as a result, a myriad of ECM changes occur during pathogenesis [18,26,36].

The ECM also has an important role in regulating synaptic function and development [37]. As the brain develops postnatally, the composition of the ECM changes to facilitate this function [38,39]. In the early stages of development, the ECM is dynamic and permissive to facilitate neuronal plasticity [40,41]. As the brain reaches the end of this critical period, which is marked by extensive neuronal outgrowth and synaptic refinement, the ECM is remodeled and replaced by an adult form enriched by PNN [30]. The PNNs are located between neurons and glia and act as a physical barrier that inhibits further synapse development [39]. The composition of the ECM is regulated by neurons and glial cells through the secretion of ECM proteases such as matrix metalloproteinases (MMPs), and a disintegrin and metalloproteinase with thrombospondin motifs (ADAMTS), which play a crucial role in ECM remodeling [42].

Brain ECM components are synthesized and secreted by both neurons and glial cells [43,44,45]. However, in the Central Nervous System (CNS), glial cells act as major regulators of the fate of the ECM [14]. Microglia, the main immune cells in the brain parenchyma, play an important role in the homeostasis of the brain ECM [46,47]. Microglia perform phagocytic removal of ECM components during synaptic remodeling [47,48,49,50]. It has been suggested that during synaptic remodeling, cell-to-cell interactions occur between microglial processes and dendrites [51], resulting in the phagocytic breakdown and remodeling of the ECM by microglia [52]. Accordingly, microglia-based clearance or modulation of the ECM around each synapse serves as the fuel to support synaptic remodeling [47], whereas microglia’s dysfunction results in aberrant ECM clearance or buildup, which contributes to the pathophysiology of the disease [14]. In animal models of PD, regions of neuronal degeneration were found to have an increased density of microglia [53]. A study on mice also reported brain injury as a promoter of the interaction between microglia and dendrites and subsequent neurotoxicity [54]. Activated microglia also cause blood–brain barrier disruption [55], which could lead to fibroblast infiltration and subsequent ECM breakdown in PD [56].

Astrocytes play a role in the elimination of extracellular α-synuclein, a protein associated with the pathogenesis of PD, and also protect neurons from the propagation of the same protein [57]. They also maintain a neuroprotective environment in the CNS under normal and inflammatory conditions by releasing a variety of ECM glycoproteins such as laminins and tenascin-C [58]. In various disease processes, reactive astrocytes can become reactive or asthenic [59], and in PD, a special inflammation process without reactive astrocytes was previously reported [60]. In addition, these glial cells help maintain the structural integrity of the blood–brain barrier, which is disrupted in PD cases [61]. The blood–brain barrier, on the other hand, is a highly regulated and dynamic structure that is additionally affected by interactions between its cellular and ECM components [62,63]. Due to their crucial role in the formation of myelin, oligodendrocytes, glial cells that produce myelin to wrap axons, also have active roles in PD [64]. The ECM forms the microenvironment that crucially controls the cellular fate of oligodendrocytes and other cells, and the proper functioning of each ECM component determines their development and pathology [65,66]. Overall, this dynamic interplay between glial cells, PD, and their surrounding milieu highlights the importance of glial cells and ECM in neuroprotective activity.

Over the past few decades, the application of high-throughput technologies has fundamentally advanced our understanding of disease mechanisms [67,68,69,70,71,72,73,74,75,76,77]. However, understanding the link between cells and the surrounding ECM remains a formidable task [26]. Many cellular activities are dominated by proteins, and knowing how these processes are controlled at the protein level is crucial for understanding the underlying molecular causes of diseases [78]. The understanding of PD pathophysiology was substantially aided by proteomic studies of brain tissue [79,80,81]. Samples from PD patients and PD animal models have displayed damage to the macromolecules of intracellular components [82,83,84,85,86,87]. Complex disease mechanisms involving neuroinflammation, oxidative stress, and the ubiquitin proteasomal system (UPS) are commonly linked with PD [88,89]. Despite the efforts to identify pathways and targets for the development of a defined therapeutic plan for PD, it remains elusive. The reason why the current understanding of PD development is incomplete could be attributed to the fact that the identified pathways and targets do not present a complete picture, which may be further exacerbated by the limited sample types and restricted brain regions considered in proteomic studies.

The lag behind diagnostic biomarkers and effective treatment can further be attributed to the unbalanced emphasis given to intracellular components, with little attention being paid to the brain ECM. The brain ECM is vital for neural plasticity and is also known to play an important role in neurodegeneration [90,91]. Despite its critical role in the regulation of cellular function, few studies have addressed it in PD [92,93,94,95]. In addition, only a small number of studies that specifically examine the ECM have been performed so far, and these investigations show that PD patients have altered ECM components [96,97]. The fact that research is concentrated on certain areas of the brain may also conceal the method by which the association between the ECM and PD is understood. As demonstrated in postmortem tissue of AD patients [98], PD may also involve numerous distinct locations, including those parts of the brain that have not been known to be impacted by the disease. It has been shown that injury or inflammation can cause the degradation of the ECM, leading to the release of various ECM molecules, including hyaluronan fragments, tenascins, and sulfated proteoglycans [24,99,100]. These fragments can travel through the extracellular space and affect adjacent regions in a paracrine manner, which can serve as a biomarker for the early diagnosis of PD as well as be used to monitor its prognosis [100]. Thus, the precise role of the ECM in PD pathology has been masked by several variables, which necessitate a comprehensive evaluation of studies that have been conducted on various brain regions and reported alterations of ECM components in PD.

While proteomics can provide a lot of information about the composition of the ECM, it does have some limitations. Some ECM proteins are highly insoluble or difficult to extract so they may not be amenable to proteomic analysis [101]. Additionally, the ECM is not composed solely of proteins; it also contains other molecules such as glycosaminoglycans, which are not always manageable for proteomic analysis [28,102]. Similarly, proteomic analysis can also be affected by post-translational modifications, including glycosylation, which can modify the identity and function of proteins [103]. The inability of current discovery strategies to detect low-abundance and transient protein species is the additional challenge of proteomic analysis to give a complete picture of ECM [102,104].

Here, we aimed to examine the differences in ECM expression and composition between PD patients and matched healthy controls, summarize key findings, and provide recommendations for the direction of future ECM-related research. To do this, we performed a thorough literature search to pinpoint the ECM proteins that are differentially expressed in PD. Accordingly, several ECM proteins including annexins, collagens, versican, and tenascins were identified. We also identified and summarized transcriptomic studies to identify genes and pathways that are differentially expressed in PD patients. These studies have reported ECM–receptor interaction, focal adhesion, cell adhesion molecules, and cell adhesion together with the integrin signaling pathway to be differentially expressed in PD. We believe that our review will elicit focused primary studies and thus support the ongoing efforts of the discovery and development of diagnostic biomarkers of as well as therapeutic agents for PD.

## 2. Methods

### 2.1. The Literature Search Strategy

All articles published in English were searched in PubMed and Google Scholar. The information was extracted from proteomic and transcriptomic studies that reported differentially expressed ECM-related proteins and genes/biological pathways. A comprehensive literature search was performed until 10 February 2023 with the search terms: “Proteomic* AND Parkinson’s disease”, “Parkinson’s disease AND Extracellular matrix”, Transcriptomic* AND Parkinson* disease AND Extracellular matrix”, “Gene expression profiling AND Parkinson’s disease”. Only studies that included postmortem/brain tissue samples and induced pluripotent stem cells (iPSCs)/neurons of human origin were included. The reference list of all identified studies was also scanned for other potentially relevant studies. Following the search, all identified citations were collated and uploaded into a citation management system. The search was re-run before summarizing the data, and additional studies retrieved were also screened for inclusion.

### 2.2. Selection Criteria

Initially, two independent reviewers screened and retrieved the articles based on the titles and abstracts, and then the full texts of the identified articles were evaluated.

#### 2.2.1. Inclusion Criteria

- Proteomic studies
- Genome-wide transcriptomic studies
- Information on differentially expressed proteins/genes/pathways related to control conditions
- Samples employed either from human patients or cell lines of human origin
- Non-review articles.

#### 2.2.2. Exclusion Criteria

- Studies conducted on nonhuman tissue or cell lines
- Interventional studies
- Literature reviews.

### 2.3. Data Extraction and Management

Two reviewers independently extracted data from the included studies using a well-structured data extraction format with strict adherence to the inclusion criteria. Extracted information included the author’s name, year of publication, number of participants, the status of the study participants (case or control), demographic characteristics (e.g., sex and age), PD type (idiopathic or genetic), sample type (brain tissue or iPSCs), brain region, post-mortem interval, proteomic/transcriptomic method, identified proteins/genes/pathways and regulations. The data extracted by the two reviewers were first compared and then merged into one datasheet. The data extraction form and all extracted data are provided as supplementary files. The EndNote X7.5 citation manager (Thomson Reuters, New York, NY, USA) was used to store, organize and manage all the references.

Every protein and gene/genomic pathway that has been reported to be altered were manually collected from both the main text and the Supplementary Materials. The proteins were organized according to their respective human Uniprot ID. Proteins, genes, and biological pathways that are commonly reported (reported by, at least, two articles) to be differentially expressed and other relevant evidence were separately presented in the Results section below. A summary table with detailed information on the included articles is presented in a supplementary file.

## 3. Results

### 3.1. Literature Search Results

**Figure 1:**
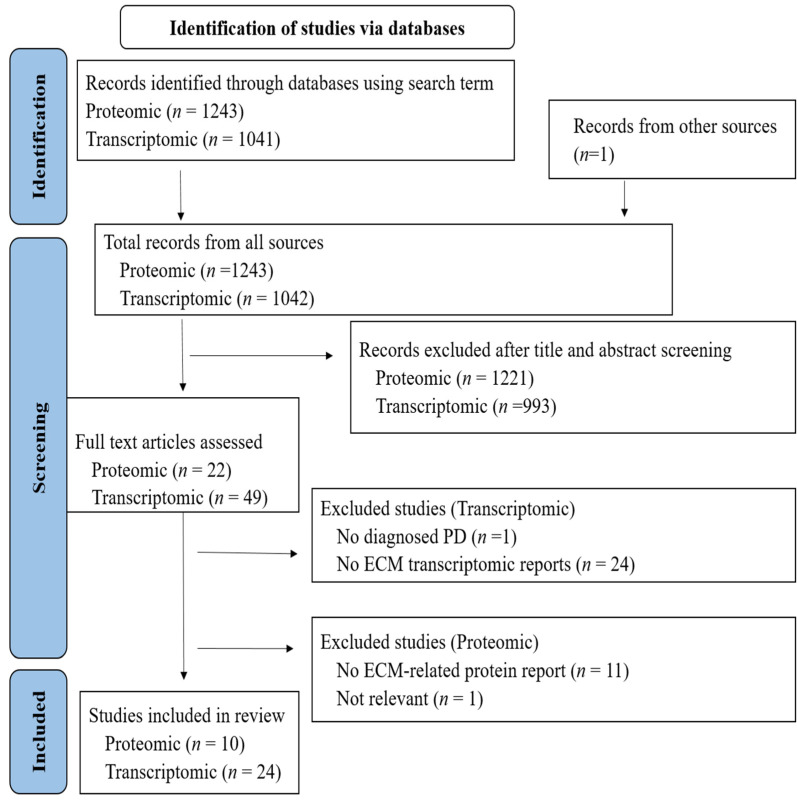
Preferred reporting items for systematic reviews and meta analyses (PRISMA) flow diagram of the search strategy.

The total number of research articles from the database and manual search for proteomic studies was 1243. Following a title and abstract screening, 22 articles were found to contain a PD control comparison and proteomic analysis. After a full-text review, only 10 articles were identified to contain an ECM protein-related report and differentially expressed ECM proteins and thus selected for data extraction. The search for transcriptomic studies identified a total of 1041 studies. These studies were screened based on the titles and abstracts, resulting in 49 articles. Then, the full texts were reviewed and 24 articles were eligible for inclusion (Figure 1).

### 3.2. Summary of the Demographic Characteristics of the Study Participants

**Figure 2:**
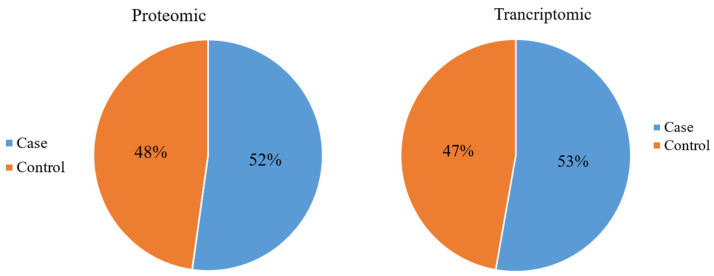
The percentage of PD patients (“case”) and controls from the included proteomic and transcriptomic studies.

**Figure 3:**
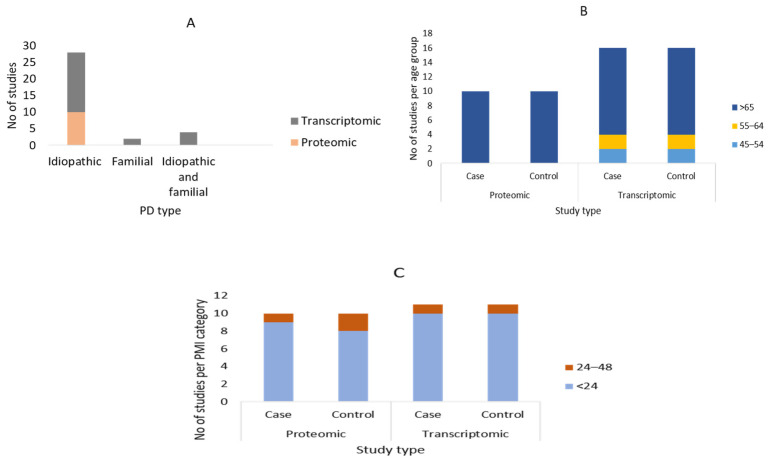
Distribution of patient- and sample-related variables of both proteomic and transcriptomic studies. Distribution of PD type (**A**); age distribution (**B**); PMI distribution of brain tissue-based studies (**C**).

All proteomic studies and the majority of the transcriptomic studies were from post-mortem brain tissue-based samples. Four articles with transcriptomic analysis were from iPSC-based studies [67,68,69,105]. One of the articles reported an analysis of post-mitotic catecholaminergic neuron-like cells, which also constitute DA neurons [106]. From the eligible studies, 247 participants (129 cases and 118 controls) were from proteomic analysis, and 1021 participants, (539 cases and 482 controls) were from transcriptomic analysis (Figure 2). All of the cases in proteomic analysis, and most of them in the transcriptomic analysis, were idiopathic PD cases (Figure 3A). The average age of the participants from both proteomic and transcriptomic studies displayed that most of the participants were aged individuals (>65 years) (Figure 3B). Most studies reported an average post-mortem interval (PMI) of less than 22 h, and in most of the proteomic studies, age, gender and PMI were matched between the case and the controls; if not, these variables were controlled (Figure 3C, Table 1).

### 3.3. Description of the Included Articles

**Figure 4:**
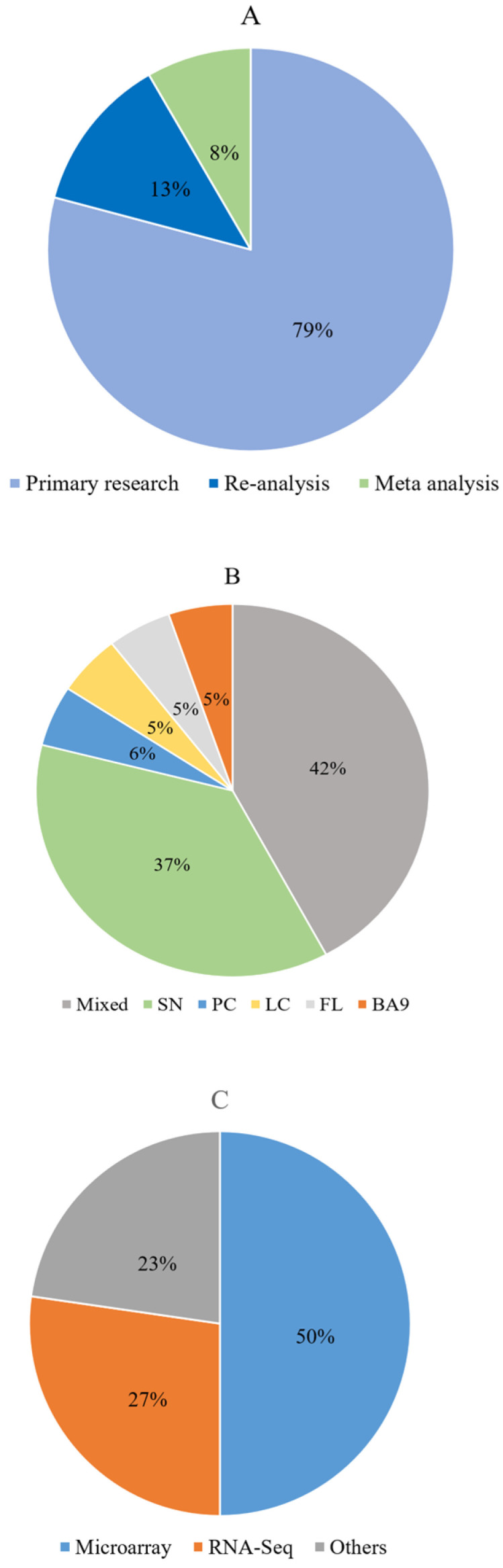

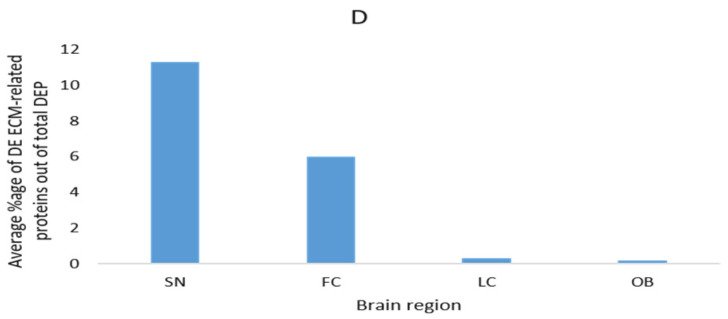
Summary of data from both proteomic and transcriptomic studies. The origin of the included articles (**A**); brain region from which the samples were taken for transcriptomic studies (**B**); methods applied for transcriptomic analysis (**C**); percentage of ECM-related differentially expressed proteins per brain region (**D**) (SN: substantia nigra; FC: frontal cortex; LC: locus ceruleus; OB: olfactory bulbs; FL: frontal lobe; PC: posterior cingulate cortex; DEP: differentially expressed protein).

All of the proteomic studies and the majority of the transcriptomic articles were from primary studies of postmortem tissue (Figure 4A, Table 2). Except in two studies [96,109], the reported number of differentially expressed ECM proteins was less than ten. The total share of differentially expressed ECM proteins reported from each proteomic study was less than one-fifth of the total differentially expressed proteins in that specific study (Table 2). Most of the articles in transcriptomic studies incorporated more than one brain region followed by the substantia nigra, which was solely used by 37% of the included articles (Figure 4B). However, the majority of the proteomic studies used samples from the frontal cortex followed by samples of the substantia nigra (Table 2), but most of the reported ECM-related differentially expressed proteins were from the substantia nigra (Figure 4D). Liquid chromatography with tandem mass spectrometry (LC-MS/MS) was the most commonly utilized method for the proteomic studies (Table 2), whereas microarray with RT-qPCR validation was the common method reported from transcriptomic studies (Figure 4C).

### 3.4. Description of Commonly Reported Differentially Expressed ECM and ECM-Related Proteins

**Figure 5:**
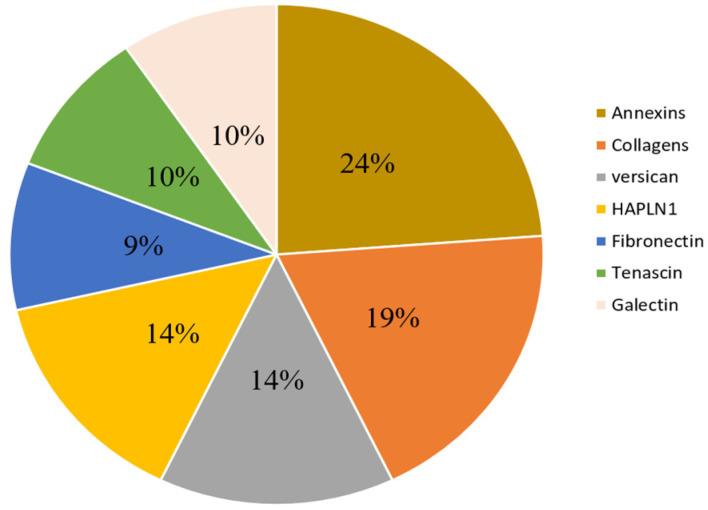
Frequency of commonly reported (reported from, at least, two of the included studies) differentially expressed proteins from postmortem tissue of PD patients compared with healthy controls.

A total of 46 proteins that are related to the brain ECM were identified from the selected proteomic studies of postmortem tissues. Some of them were reported in more than one study, resulting in 33 unique proteins. Annexins and collagens were the most commonly reported proteins followed by the versican core protein and brain link protein (Figure 5). Most of the ECM-related proteins were reported to be upregulated. All collagen and tenascin subunits were identified to be upregulated. However, different studies reported hyaluronan, proteoglycan link proteins, and fibronectin differently (as dysregulated in both directions) (Table 3).

### 3.5. Description of Commonly Reported Differentially Expressed ECM and ECM-Related Gene Groups/Pathways/Processes

**Figure 6:**
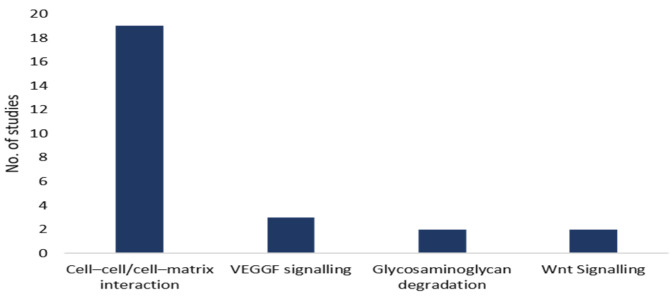
Commonly reported pathways/processes/groups of genes from postmortem and iPSC-based studies (Cell–cell /cell–matrix = ECM–receptor interaction, cell–matrix interaction, focal adhesion, cell adhesion molecules and cell adhesion).

**Figure 7:**
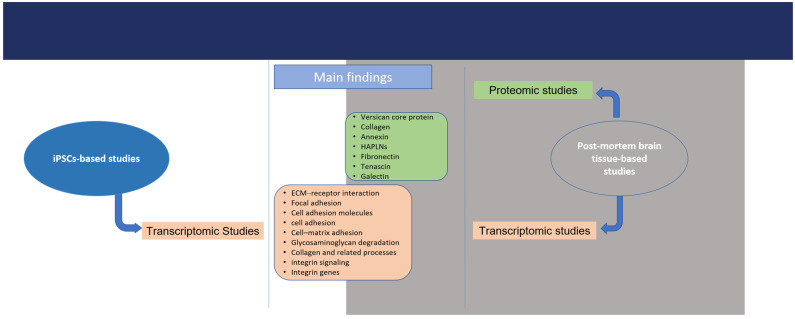
A diagram showing the main findings from all studies included in the review (the color of the study type is matched with the listed main finding; the gray background is for postmortem brain tissue-based studies and the white background is for iPSC-based studies, orange color for transcriptomic studies with related findings and light green for proteomic studies with related findings).

From the transcriptomic studies of postmortem tissues and iPSC-based studies, focal adhesions were the most commonly reported macromolecular assemblies followed by cell adhesion molecules and cell adhesion. ECM–receptor interaction and *VEGF* signaling were equally reported by three articles (Figure 6). ECM–receptor interaction, cell adhesion molecules, glycosaminoglycan degradation, and integrin signaling were all reported as upregulated across the studies, and collagen and related processes were reported to be downregulated. The remaining gene groups/pathways/biological processes were reported to be dysregulated in both directions. Similarly to integrin signaling, all integrin-related genes reported in the articles were upregulated, whereas collagen-related genes were reported to be dysregulated in both directions (Table 4). Some of these pathways are categorized into one (cell–cell/cell–matrix) and presented together with other dysregulated pathways as given below (Figure 6 and Figure 7).

## 4. Discussion

According to our search result, a few studies reporting differentially expressed ECM components were identified, and even fewer studies were identified that focused on proteomic studies related to the ECM. Furthermore, the accessed studies reported a relatively small number of ECM-related genes and proteins that are altered in PD compared to total number of dysregulated genes. The frontal cortex and substantia nigra were the most frequently sampled regions, with collagens, annexins, tenascins, and versican being the most commonly reported proteins. The transcriptomic studies identified several differentially expressed ECM-related pathways, including ECM–receptor interaction, focal adhesion, cell adhesion molecules, and cell adhesion. However, the limited number of ECM-targeted studies highlights the need for more targeted research to gain a better understanding of potential alterations in the brain’s ECM in relation to PD. The fact that only a relatively small number of ECM-related genes and proteins have been reported to be altered in PD compared to the total number of dysregulated genes reported may suggest that the role of ECM in the development of PD is only partial, despite the abundance of ECM-related genes present in the Matrix database; alternatively, it could indicate that conducting functional studies, rather than solely focusing on “omics,” is a more plausible approach for better understanding the contribution of the ECM to PD pathologies. Overall, the compiled evidence from our review work could potentially enhance our comprehension of the pathophysiology of PD, and also help to guide ongoing efforts towards identifying reliable molecular markers and effective interventions to halt its progression.

Around one-third of the ECM is made up of collagens, but there is little data to show how they are affected in PD [129]. Despite this barrier, several types of collagens from different categories—fibril-forming (I), network-forming (IV) and beaded filament-forming (VI)—were observed to be differentially expressed in PD patients compared with matched controls. In our analysis above, we showed the dysregulation of different collagen proteins and genes from brain tissue and iPSC-based human studies. Similar findings have also been reported from in vitro studies, animal PD models, and PD patients. In a 3D cell culture of primary rat cortical neurons, Cullen et al. [130] observed an association between type IV collagen, the major protein component of the basement membranes, and neurite outgrowth. Transgenic mice with alpha-synuclein overexpression also exhibited elevated type IV collagen expression, implying a potential correlation between alpha-synuclein accumulation and basement membrane dysfunction in PD [131]. Type VI collagen is mainly found in the connective compartments of the CNS, and is known to interact with other ECM components [132]. An analysis of animal brain sections revealed that a deficiency in collagen VI accelerates neurodegeneration by inhibiting autophagy and inducing apoptosis [133]. An additional study on transgenic mice has also demonstrated its neuroprotective role against the toxicity of amyloid-β peptides and UV-induced damage [134]. Furthermore, a study on patients with loss-of-function mutations in type VI collagen has also linked this protein to dystonia, a movement disorder characterized by persistent or sporadic muscle spasms [135]. Jin et al. [136], in their recent study on sporadic PD patients, reported a possible connection between the *COL6A3* gene variants and susceptibility to PD. According to our proteomic review work, collagens IV and VI were found to be upregulated in PD patients [96,97,112], although transcriptomic studies showed dysregulation of collagen IV in both directions [67,68]. Along with other studies from different PD models, our review work highlights the functional role of collagen (especially collagen VI) in neuronal cells and their neuroprotective potential against neurodegeneration [137].

The key perineuronal net (PNN) components, such as lecticans, tenascin R, and link proteins, interact with one another to form the PNN’s molecular framework, which wraps around perikaryon and proximal dendrites of certain nerve cells [138]. Among the lecticans, brevican, neurocan, versican and other types of proteoglycans such as decorin were reported to be differentially expressed in PD in the proteomic studies included in the current review work [96,97,107,109]. However, only the versican protein was widely reported among them and observed to be upregulated across the studies [96,97,107]. Versican is a non-fibrous component of the brain’s ECM, acting as a core protein to which side chains of carbohydrates bind to create proteoglycans [28]. It is a multifunctional protein modulating cell adhesion, migration, and inflammation, thereby interacting with immune cell receptors and also other ECM components such as fibronectin and tenascin [139,140,141]. The binding of immune cells to the versican–ECM complex may break down the ECM, leading to neuroinflammation and apoptosis [141,142]. However, whether the intact or fragmented versican is responsible for neuroinflammation and apoptosis needs further investigation. According to Downs et al. [97], its alteration involves both proteomic and glycoproteomic changes in PD, and they emphasized the importance of changes in its glycosylation pattern on the inflammatory process in PD. Overall, further research into its neuroinflammatory mechanism and targeted work could lead to a novel approach to treating PD.

According to the reports from proteomic studies included in the current review, fibronectin and tenascin were also among the widely reported dysregulated ECM proteins [96,97,109,110]. Tenascins (C and R) were demonstrated to be upregulated, but mixed results were observed in the case of fibronectin [97,109]. Such opposing expressions of different glycoproteins were also implicated in multiple sclerosis [143]. Like in PD, high tenascin levels were also reported in the brains of Alzheimer’s disease (AD) patients [138]. According to a study conducted on multiple sclerosis patients, this enhanced production may probably represent a defensive mechanism, yet excessive production could lead to disorganized matrix depletion and the suppression of restorative activities [28,144]. Additional studies on an in vitro model of induced inflammation of hippocampal neurons co-cultured with glial cells and on an AD mouse model showed that the inhibition of tenascin’s function and compounds that reduce its production suppresses neurodegeneration [145,146]. These outcomes in other neurodegenerative diseases and their upregulation in PD patients indicate the importance of tenascins as a potential therapeutic target for neurodegenerative diseases. To stabilize the ECM at the cell surface, fibronectin needs uninterrupted polymerization into fibrils, which in turn requires the adequate delivery of integrins [147,148]. In our literature review, integrin gene expression was upregulated in both postmortem and iPSC-based studies of PD patients [67,68] and the opposite expression between fibronectin and integrin was also reported in iPSC-based studies [67,68]. Such contradicting results from co-functioning genes necessitate further work to figure out their exact contribution to PD pathogenesis.

Annexins are another group of proteins reported to be differentially expressed in postmortem tissue of PD patients. According to a report based on affinity chromatography and solid phase assays, these proteins are known to bind with glycosaminoglycans (GAGs), ECM components, with specific binding affinities [149]. In this review work, annexin A6 was downregulated while annexins A1, A2, and A5 were upregulated [97,109,111,114]. It has been demonstrated that annexin A6 acts as a recognition component for GAGs in the extracellular space [149]. In contrast to annexin A6, transcriptomic postmortem studies from our review work indicated the upregulation of the GAG degradation pathway [123,124]. Such opposing expressions may likely indicate the disruption of their molecular networks and associated signaling pathways in PD [149]. Furthermore, it has been demonstrated that, in conjunction with annexin A2, annexin A6 interacts with tau, which is thought to contribute to the pathological redistribution of tau in Alzheimer’s disease. [150]. Recombinant human annexin A1 (*hrANXA1*) was demonstrated to lower amyloid-β levels in an AD mice model [151] whereas annexin A5, whose cerebrospinal fluid level was reported to match disease severity in AD patients, was implicated as a biomarker in AD [152]. These pieces of evidence together imply the impact of annexins in neurodegeneration and their potential as biomarkers and therapeutic targets.

Along with proteomic study, transcriptomic study is also providing valuable insights into the genetic underpinnings of PD and paving the way for new and personalized approaches to its diagnosis and treatment. Consequently, we gathered literature reporting transcriptional alterations in the ECM of PD patients. Several pathways and processes, including ECM–receptor interaction, focal adhesion, cell adhesion molecules, and cell adhesion were observed to be dysregulated after gene ontology (GO) analysis. Focal adhesions are the specialized cell adhesion structures that mediate the interaction between the ECM and the intracellular actin cytoskeleton [153,154]. Cell adhesions occur through the interactions between cell adhesion molecules (CAMs) and transmembrane proteins located on the cell surface, which connect cells to the ECM [155]. These interactions involve two types of receptors, cadherin and integrin receptors, which mediate cell–cell and cell–ECM adhesion, respectively [156,157]. In the ECM, integrin binds with laminins, cell adhesion molecules, and major components of the basement membrane [158]. From this review work, both integrins and laminins were observed to be upregulated in PD [67,68,113,120,126,127,128]. Furthermore, the significance of cell adhesion for cell survival and physiology highlights the importance of proper communication between the ECM and integrins [159]. This evidence, together with the dysregulation of their signaling pathways and individual genes in PD, underlines integrins as a potential and valid target molecule for PD treatment. Previous success in developing integrin-targeted antibodies blocking ligand binding [160,161] and downstream signaling [160,162] further support the significance of integrins as important drug targets.

According to the included studies, the substantia nigra, the primary region of the brain involved in PD, has a relatively larger average percentage of differentially expressed ECM proteins per total number of differentially expressed proteins compared to other brain regions. Overall, most of the proteomic studies from our review reported a small number of differentially expressed ECM proteins. Several factors including the quality of postmortem human samples and methods applied for sample dissociation and extraction may affect protein extraction [163]. The postmortem interval (PMI) is one of the important parameters in postmortem studies, particularly when evaluating postmortem tissue sample quality. There has been evidence that a prolonged PMI causes protein breakdown, which substantially reduces the amount of detectable protein during subsequent tissue processing [163]. However, if the autopsy is taken as soon as possible (PMI < 22 h), as demonstrated, protein integrity will be retained, which is consistent with the majority of the articles included in our review work. In most of the articles the age, gender, and PMI were reported to be matched/controlled. Therefore, other variabilities between individual patients and overall health at the time of death may be more likely here [163,164].

Despite the evidence of hyaluronan remodeling in the neurodegeneration mouse model [50,165], in the studies that we analyzed, it was not shown to be dysregulated. However, one of the studies reported the upregulation of the heparan sulfate-glucosamine 3-sulfotransferase 2 (*HS3ST2*) gene in postmortem brain tissue [120]. In addition to this, changes in the glycosylation profile of versican were also reported in PD [97]. Versican plays a role in neuroinflammation by mediating communication between neutrophils and cytokines [166]. The glycosylation pattern of versican affects this process, and aberrant expression and glycosylation could potentially disrupt this inflammatory process in PD. Another study included in the analysis also reported the changes in brain tissue associated with glycosaminoglycans (GAGs) and proteins during normal aging and compared them to those seen in PD. The study found that the changes in the composition of heparan sulfate disaccharides that occur during human aging are different from those seen in PD [96]. These alterations in the disaccharide profiles of GAGs that occur in PD could be indicators of changes in the way that signaling proteins are presented to cellular receptors and the inadequacy of proteomic studies to present complete pictures of the ECM’s status. Such changes in ECM components have important implications for PD pathogenesis. Studies have also shown that analysis of ECM proteins and carbohydrates during injury may provide insight into the underlying pathobiology of PD [100]. Along with its abundance, a disruption of the ECM might thus be a signal of tissue damage and its components could potentially serve as biomarkers to diagnose or monitor PD. The samples that are used for omics analysis could also harbor inherent genetic, proteomic, and glycomic variability [96]. However, an integrated analysis of multi-omics data would possibly give replicable results and minimize the time lapse in bringing bench work to the bedside.

Some of the included iPSC- based studies were conducted on both familial and idiopathic PD [67,68]. These studies suggest the biological origin of even idiopathic PD. However, this might be confounded by the fact that the samples that are used for omics analysis could also harbor inherent genetic, proteomic, and glycomic variability [96]. In general, studying the ECM presents several challenges due to its complexity and dynamic nature. Standardized methods, proteomic techniques, and better in vitro models will be essential for advancing our understanding of ECM dynamics and its role in tissue homeostasis and disease. Maintaining its complex three-dimensional nature is one of the challenges in 2D iPSC cultures. This can make it difficult to compare results across studies and limit the reproducibility of findings. However, integrated analysis of multi-omics data would possibly give replicable results and minimize the time lapse in bringing bench work to the bedside. Importantly, in recent years, organoids, which are 3D scaffolds that mimic the architecture and functionality of organs *in vivo*, have been developed [167,168,169]. These offer a powerful tool for studying brain diseases and disorders [170,171,172]. Brain organoids may therefore be used for modeling the development, maturation, and aging of the brain ECM, specifically in PD. However, Matrigel is frequently employed for the embedding of the embryoid bodies (EBs) during the production and expansion of almost all kinds of organoids [168,173]. Since Matrigel’s main components are collagens and laminin, it introduces a confounding factor when trying to model the brain ECM, and this is an important issue that needs to be addressed, perhaps by using other types of gels with a similar strain modulus [167,174,175].

In conclusion, limited relevant studies were accessed from our search, indicating that much work remains to be carried out to better understand the roles of the ECM in neurodegeneration and PD. Our work summarizes proteomic and transcriptomic studies of ECM genes and proteins that are dysregulated in PD. In general, compared to the total number of dysregulated proteins and genes, only a few ECM-related proteins and genes were identified. From the collective evidence, we observed that, although the current knowledge on the involvement of aberrant ECM proteins in PD is still in its infancy, it is clear that changes in the expression of ECM macromolecules play important roles in PD. The most commonly reported differentially expressed proteins were annexins, collagen VI, versican, and tenascins, whereas ECM–receptor interaction, focal adhesion, cell adhesion molecules, and cell adhesion, as well as the integrin signaling pathway and individual integrin genes, were commonly dysregulated at the transcription level. These ECM components and pathways are potential sites to be investigated, validated, and used as drug targets for PD treatment.

## Figures and Tables

**Table 1:** Patient and sample information from the included proteomic studies.

No. of Study Participants		Average Age		Sex		PMI (h)		Remark	Ref.
Case	Control	Case	Control	Male	Female	Case	Control		
6	6	77.8	77.8	0	12	5.75	7.15	Matched *	[107]
3	3	81.7	83.3	6	0	19.7	20		[108]
3	3	79	72.7	3	3	18.7	24		[109]
20	5	-	-	-	-	<12 h	<12 h	Matched *	[110]
28	37	77.6	68	28	37	10.25	14.1	Matched */cohort	[96]
28	36	77.6	68	28	36	10.25	14.1	Matched */cohort	[97]
5	5	84.2	77.4	6	4	35.6	30.2	Matched *. PMI controlled	[111]
21	8	79.9	77.8	17	12	10.6	17.4		[112]
12	12	76.8	79.5	24	0	4.16	5.66	** controlled	[113]
3	3			4	2			Matched *	[114]

* Matched = age, gender and PMI are matched; ** controlled = age and PMI are controlled.

**Table 2:** Differentially expressed proteins from postmortem brain samples and the methods applied.

Brain Region	Method	DEP	DEP (ECM-Related)	DEP (ECM-Related) %	Ref.
Locus ceruleus	LC-MS	87	2	1.1	[107]
Substantia nigra	MS/MS	23	2	8.7	[108]
Substantia nigra	LC-MS/MS	204	12	5.9	[109]
Frontal cortex (middle frontal gyrus)	LC-MS/MS	200	2	1	[110]
Frontal cortex Brodmann area 9	Q-Extractive HF MS	89	14	15.7	[96]
Frontal cortex Brodmann area 9	Q-Extractive HF MS	112 *	8	7.1	[97]
Substantia nigra	LC-MS/MS	11	2	18.2	[114]
Substantia nigra	2D-GE, MS/MS	16	2	12.5	[111]
Olfactory bulbs	LC-MS/MS	168	1	0.6	[112]
Frontal cortex Brodmann area 9	LC-MS/MS	283	1	0.2	[113]

* The total ECM-related components identified; DEP = differentially expressed proteins.

**Table 3:** Commonly reported differentially expressed ECM-related proteins from postmortem tissue of PD patients compared with healthy controls (reported from, at least, two of the included studies).

Components	Protein Names	Gene Names	Up/Down	Ref
Versican family	Versican core protein	*VCAN*, *CSPG2*	up	[96,97,107]
Collagen family	Collagen alpha-1(I) chain	*COL1A1*	up	[96,97]
	Collagen alpha-2(I) chain	*COL1A2*	up	[96,97]
	Collagen alpha-1(IV) chain	*COL4A1*	up	[96]
	Collagen alpha-2(IV) chain	*COL4A2*	up	[96,97,112]
	Collagen alpha-3(VI) chain	*COL6A3*	up	[96,97]
Annexin family	Annexin A1	*ANXA1*	up	[109,114]
	Annexin A2	*ANXA2*	up	[97,109]
	Annexin A5	*ANXA5*, *ANX5*	up	[97,111]
	Annexin A6	*ANXA6*, *ANX6*	down	[97,108]
Hyaluronan and proteoglycan link protein family	Hyaluronan and proteoglycan link protein 1	*HAPLN1 (CRTL1)*	up	[96]
	Hyaluronan and proteoglycan link protein 2	*HAPLN2*	Down and up	[96,109,114]
	Hyaluronan and proteoglycan link protein 4	*HAPLN4*	down	[109]
Fibronectin family	Fibronectin (FN)	*FN1 (FN)*	Up and down	[97,109]
Tenascin family	Tenascin, TN	*TNC (HXB)*	up	[96]
	Tenascin-R (TN-R)	*TNR*	up	[110]
Galectin family	Galectin-1, Gal-1	*LGALS1*	up	[111]
	Galectin-3, Gal-3	*LGALS3*, *MAC2*	Down	[107]
	Galectin-3-binding protein	*LGALS3BP M2BP*	up	[109]

**Table 4:** Commonly reported differentially expressed ECM-related gene groups/pathways/biological processes from iPSCs and postmortem tissue-based studies of PD patients compared with healthy controls (reported from, at least, two of the included studies).

Genes/Gene Groups/Pathways/Biological Processes	Sample	Up/Down	References
ECM–receptor interaction	Brain tissue, post-mitotic catecholaminergic neuron-like cells, iPSC-derived DA neurons	up	[68,106,115]
Focal adhesion	Brain tissue and iPSC-derived DA neurons	up	[68,115,116,117,118]
	iPSC-derived DA neurons	down	[67]
Cell adhesion molecules	Brain tissue and iPSC-derived DA neurons	up	[67,115,117,119]
Cell adhesion	Brain tissue	up	[113,120,121]
	iPSC-derived DA neurons	Down	[69]
Cell–matrix adhesion	Brain tissue	up	[115]
	Brain tissue	down	[122]
Glycosaminoglycan degradation	Brain tissue	up	[123,124]
Collagen and related processes	iPSC-derived DA neurons	down	[67,69]
Integrin signaling	Brain tissue	up	[113]
***ITGA1, ITGA 3, ITGA 4, ITGA 5, ITGA 7, ITGA 11, ITGAM, ITGB3BP***	Brain tissue and iPSC-derived DA neurons	up	[67,68,120]
***COL1A2, COL4A1, COL4A2,*** ***COL6A3, COL12A1***	iPSC-derived DA neurons	down	[67,125]
***COL1A2, COL4A1, COLA2, COL18A1***	Brain tissue and iPSC-derived DA neurons	up	[68,120]
***LAMA1, LAMA2, LAMB1, LAMB2***	Brain tissue and iPSC-derived DA neurons	up	[67,120,126,127,128]
***LAMA3***	Brain tissue	Down	[126]

## Supplementary Materials

The supporting information can be downloaded at: https://www.mdpi.com/article/10.3390/ijms24087435/s1.
